# A comparison of two emergency medical dispatch protocols with respect to accuracy

**DOI:** 10.1186/s13049-017-0464-z

**Published:** 2017-12-29

**Authors:** Klara Torlén, Lisa Kurland, Maaret Castrén, Knut Olanders, Katarina Bohm

**Affiliations:** 1 Department of Clinical Science and Education, Karolinska Institutet, Södersjukhuset, SE 118 83 Stockholm, Sweden; 2 Department of Medical Sciences, Örebro University and Department of Emergency Medicine, Örebro University Hospital, Örebro, Sweden; 3 Department of Emergency Medicine and Services, Helsinki University Hospital and Helsinki University, Helsinki, Finland; 4 Department of Anaesthesiology and ICU, Lund University Hospital, Lund, Sweden; 5 Department of Emergency Medicine, Södersjukhuset, Stockholm, Sweden

**Keywords:** Emergency medical dispatch, Emergency medical services, Dispatch protocol, Medical order entry systems, Patient safety

## Abstract

**Background:**

Emergency medical dispatching should be as accurate as possible in order to ensure patient safety and optimize the use of ambulance resources. This study aimed to compare the accuracy, measured as priority level, between two Swedish dispatch protocols – the three-graded priority protocol Medical Index and a newly developed prototype, the four-graded priority protocol, RETTS-A.

**Methods:**

A simulation study was carried out at the Emergency Medical Communication Centre (EMCC) in Stockholm, Sweden, between October and March 2016. Fifty-three voluntary telecommunicators working at SOS Alarm were recruited nationally. Each telecommunicator handled 26 emergency medical calls, simulated by experienced standard patients. Manuscripts for the scenarios were based on recorded real-life calls, representing the six most common complaints. A cross-over design with 13 + 13 calls was used. Priority level and medical condition for each scenario was set through expert consensus and used as gold standard in the study.

**Results:**

A total of 1293 calls were included in the analysis. For priority level, *n* = 349 (54.0%) of the calls were assessed correctly with Medical Index and *n* = 309 (48.0%) with RETTS-A (*p* = 0.012). Sensitivity for the highest priority level was 82.6% (95% confidence interval: 76.6–87.3%) in the Medical Index and 54.0% (44.3–63.4%) in RETTS-A. Overtriage was 37.9% (34.2–41.7%) in the Medical Index and 28.6% (25.2–32.2%) in RETTS-A. The corresponding proportion of undertriage was 6.3% (4.7–8.5%) and 23.4% (20.3–26.9%) respectively.

**Conclusion:**

In this simulation study we demonstrate that Medical Index had a higher accuracy for priority level and less undertriage than the new prototype RETTS-A. The overall accuracy of both protocols is to be considered as low. Overtriage challenges resource utilization while undertriage threatens patient safety. The results suggest that in order to improve patient safety both protocols need revisions in order to guarantee safe emergency medical dispatching.

## Background

Emergency medical dispatching is the first link in the chain of Emergency Medical Services (EMS), and constitutes the basis for further medical assessment and treatment [1, 2]. Hence, the assessment of the emergency medical call should be as accurate as possible with respect to priority level in order to ensure patient safety and optimize the use of ambulance resources.

Different emergency medical dispatch protocols have evolved in order to assist call takers in assessing and prioritizing emergency medical calls [3]. Two of the most used dispatch concepts are Medical Priority Dispatch (MPD) [4] and Criteria Based Dispatch (CBD) [5–7]. However, there is no consensus on which individual protocol is superior or should be recommended [8–11]. Dispatching is performed in accordance with the CBD-protocol Medical Index in the majority of county councils in Sweden [12, 13]. Despite being in use since 1997, little research is available on the accuracy of the Medical Index [14, 15].

A prototype for a new dispatch protocol was developed as the result of a strategy for alignment between the Emergency Medical Communication Centre (EMCC) and the rest of the EMS [16]. The prototype, RETTS-A, is based on the triage scale Rapid Emergency Triage and Treatment System (RETTS), which is currently used in most of the Swedish emergency departments as well as ambulances [16, 17], and was adapted to the context of dispatching. However, the RETTS-A prototype for dispatching has not yet been evaluated. In summary, given the sparse research, neither of the protocols can be considered validated. The aim of the current simulation study was to compare the accuracy, measured as correctly assigned priority level, between the two Swedish dispatch protocols – the Medical Index, currently in use, and the newly developed RETTS-A.

## Methods

### Study design

A randomized controlled non-blinded simulation study was performed at the Emergency Medical Communication Centre (EMCC) in Stockholm, Sweden, between 27-10-2015 and 17-03-2016. It was designed based on a feasibility study conducted in the spring of 2015 that tested the design and realization of the current study. Data from the feasibility study is not included in the current study. The conclusion was that the designed method was working and feasible.

### Study setting

In Sweden, the single emergency number 112 and the integrated emergency services are run by SOS Alarm, a company owned by the Swedish Government and the Swedish Association of Local Authorities and Regions. All emergency calls are received by a telecommunicator, and ensure coordinated actions by the police, ambulance and other rescue services. Serving the entire of Sweden, thirteen EMCCs handle approximately three million calls per year. One third of these are emergency medical calls. Emergency medical dispatching is operated by SOS Alarm in eighteen of Sweden’s twenty-one counties [18]. The remaining counties operate their own emergency medical dispatching. Emergency calls made from these counties via 112 are first answered at any of SOS Alarm’s EMCCs. In case of a medical emergency, the calls are directed to respective counties dispatch central. The telecommunicator assesses and prioritizes the emergency medical call. The dispatcher coordinates the ambulance fleet, allocating an ambulance to the location in accordance with the priority set by the telecommunicator [13, 18]. The telecommunicator can be a registered nurse (RN) or a person without a formal medical education. Educational requirements for telecommunicators include a high school diploma, fluency in Swedish and English in addition to the ability to collaborate, provide service and cope with stress. All telecommunicators undergo six months of training and certification. Re-certification is required annually.

Medical Index is a three-graded priority protocol. It contains 30 chapters based on the main complaint, e.g. a symptom, a body part, a special condition or accident [12]. Each of these chapters contains listed medical conditions divided into priority levels. The telecommunicator assigns a priority level from 1 (the most urgent) to 3 (least urgent) based on the assessed medical condition. A fourth level is assigned to callers not requiring medical assistance but transportation.

RETTS-A, still a prototype, is based on the triage scale RETTS and modified to fit the complex situation of the EMCC [16]. As the original triage scale, RETTS-A is a four graded priority protocol that consists of an algorithm for vital signs and flowcharts for “emergency signs and symptoms” (ESS). RETTS include five parameters in the vital signs algorithm; airway, breathing, circulation, mental status, and environment/body temperature. Since an emergency medical call does not allow for direct interaction between the telecommunicator and the caller the vital signs “circulation” and “body temperature” were excluded. These vital signs were considered difficult to evaluate without seeing and examining the patient, and the vital signs “breathing” and “mental status” was decided to work as surrogate signs. The ESS-flowcharts each regard a specific symptom/disorder such as “affected breathing” or “abdominal/urinary tract disorders”. RETTS contains 99 flowcharts. The prototype RETTS-A, contained 32 ESS at the time of the study.

Initially, the telecommunicator is obliged to determine the status of the caller’s vital signs. The vital signs are assessed on a two to four graded scale, depending on what vital sign that is being assessed. Thereafter, the telecommunicator proceed by asking the caller about their signs and symptoms and medical history. The telecommunicator can type in words describing the presented symptoms and several suggestions on appropriate ESS shows up. The telecommunicator then assigns one or two appropriate ESS’s, based on their assessment of presenting symptoms and medical history. Like the original RETTS, the vital signs and ESS’s in RETTS-A include a grading that generates a color coded priority level from red (most urgent) to orange, yellow and green (least urgent). The final assigned priority level is the most urgent level as obtained from the vital signs and ESS’s.

### Study material

Telecommunicators working at SOS Alarm volunteered to participate. They were informed about the study and gave consent to their participation through registration on the SOS Alarms web-site. All participating telecommunicators were educated and trained in the Medical Index protocol in accordance with SOS Alarm’s guidelines. None of them had previously worked with RETTS-A. Educational material on RETTS-A was sent to the telecommunicators two weeks prior to the study. The educational material included a list of clinical expressions used in the RETTS-A’s ESS-flowcharts, that telecommunicators without health care education were not expected to be familiar with. A 30-min educational meeting was held at the start of each simulation session. Data on each telecommunicator regarding demographics, education and work experience at SOS Alarm was collected. Standard patients, i.e. persons trained to simulate a patient in a standardized way [19], were recruited from a group linked to the hospital Södersjukhuset AB, Stockholm, Sweden. The standard patients simulated the emergency medical calls, based on manuscripts described below, that were handled by the participating telecommunicators. Manuscripts for 26 patient scenarios, based on actual recorded calls extracted from the SOS Alarms database, were constructed by the authors. The scenarios represented the six most common complaints (affected breathing, chest pain, minor trauma/wound, stroke, abdominal/urinary tract symptoms and vague/undefined problems) presenting to the EMCC. Each manuscript included information on the caller’s sex, name, age and address. If the callers were not the affected person themselves, this was stated in the manuscript. An opening statement with a brief summary of the chief complaint was written for each scenario to be provided by the standard patient. Additional information to be revealed by the standard patient, if requested by the telecommunicator, included a further description of symptoms and vital parameters, time of onset, duration, activity during the call, previous medical history and medication. The manuscripts were sent out to the enrolled standard patients two weeks prior to study start.

Priority level and medical condition for each scenario was predetermined through expert consensus prior to study start. The expert group was composed of eight people with medical and operational expertise within EMCCs, hospital and pre-hospital emergency medicine. Each expert was asked to assign the most accurate priority level and medical condition based on the information provided in the manuscript in accordance with the Medical Index and RETTS-A, blinded to the other experts. The written assessments were assembled by the authors. Consensus on priority level and medical condition for each scenario was reached through two consecutive telephone conferences and one physical meeting. The consensus level was used as the gold standard in the study.

### Simulations

The simulations were performed at the test-center of Stockholm’s EMCC, using the same computers and technical equipment that are used in the operational center. A cross-over design with 13 + 13 calls was used for each session. Two telecommunicators assessed the 26 scenarios simultaneously in one simulation session. Two standard patients acted as emergency medical callers in each session. The standard patients made their calls in accordance with a manuscript, and from a location separate from the telecommunicators’. The telecommunicators were randomized to assess the first 13 calls with either Medical Index or RETTS-A, and shift to the other protocol for the remaining 13 calls. The standard patients were blinded to the protocol used by the telecommunicator. The telecommunicators were blinded to the other’s prioritization. The order of the scenarios was decided beforehand and by the research group, so that none of the six main complaints (affected breathing, chest pain, minor trauma/wound, stroke, abdominal/urinary tract symptoms and vague/undefined problems) was presented more than two calls in a row. This was done as to prevent learning effects. The same loop of scenarios was repeated for each simulation session during the entire study.

### Data collection

Data from the recorded simulated emergency medical calls were extracted from the software system at SOS Alarm and provided to the research group. Data included the unique id-number for each call, id-number for the telecommunicator handling each call, the dispatch protocol used and the assigned priority level and medical condition as well as the caller’s name, personal identification number and contact details and additional information registered by the call taker such as a detailed description of symptoms, medical history and medications.

### Statistical analysis

A power calculation was performed anticipating a difference of eight percentage points in accuracy of priority level between the two protocols. The power calculation revealed a need for *n* = 2 × 650 = 1300 unique calls (two-sided alpha 0.05, beta 0.80). The power calculation was made after the feasibility study but prior to the current main study. Accuracy in priority level for the two dispatch protocols was calculated as the number of emergency medical calls assigned with the correct priority level, i.e. in accordance with expert consensus. Sensitivity and specificity stratified by priority level as well as negative- and positive predictive value (NPV, PPV) and proportion of total over- and undertriage for each protocol were calculated. Because the two protocols have different numbers of grading an alternative analysis was performed where RETTS-A was converted from a four-graded to a three-graded priority protocol. The conversion was made by merging the two highest priority levels into one single priority level (red + orange). As secondary outcome, the number of emergency medical calls assigned the correct medical condition, was calculated. For RETTS-A, the assignment was regarded as correct if any of the two assigned ESS’s was in accordance with the expert consensus.

For continuous variables, a median and inter quartile range (IQR) was used. For categorical variables, proportions were reported with 95% confidence intervals (95% CI) and Chi2-test was used for comparisons. P-levels <0.05 were regarded as significant. All analyses were performed using SPSS statistical software, version 24.

### Ethical considerations

The current study has been approved by the Swedish Regional Ethical Board in Stockholm, dnr 2015/1637–31/5.

## Results

**Fig. 1:**
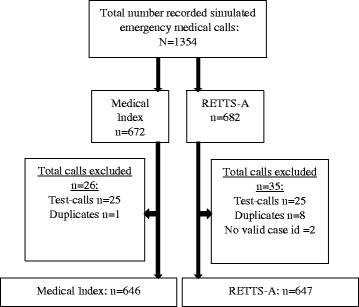
Flow chart illustrating calls included in the final analysis and reasons for exclusion

Of the aimed 1378 simulations, 24 were not performed due to technical and logistical problems. In total, 1293 of the 1354 recorded simulated emergency medical calls were included in the final analysis (Fig. 1). Six hundred and forty-six calls were assessed with the Medical Index and 647 calls were assessed with RETTS-A. Priority level was not assigned to 12 calls for the Medical Index and three calls for RETTS-A, and these calls were, therefore, excluded in the analysis of priority level. Six calls in the Medical Index group were assessed as callers not requiring medical assistance, but rather transportation and, therefore, transformed into priority level 3 in the analysis of priority level.

Three hundred and forty-nine (55.0%) of the simulated calls were assessed correctly with the Medical Index and *n* = 309 (48.0%) with RETTS-A (*p* = 0.012) (Table 1). Sensitivity and specificity were stratified by priority level, and are presented in Table 2. The sensitivity was 82.6% (95% confidence interval: 76.6–87.3%) for the highest priority level in the Medical Index and 54.0% (44.3–63.4%) for RETTS-A. Overtriage, defined as the assigned priority level being higher than the gold standard, was 37.9% (34.2–41.7%) in the Medical Index and 28.6% (25.2–32.2%) in RETTS-A (Table 3). The corresponding proportion of under-triage, defined as the assigned priority level being lower than the gold standard, was 6.3% (4.7–8.5%) in Medical Index and 23.4% (20.3–26.9%) in RETTS-A (Table 3).

**Table 1:** Total accuracy in priority level using Medical Index and RETTS-A (*n* = 1278)

	Dispatch protocol	
	Medical Index (*n* = 634)	RETTS-A (*n* = 644)
Calls assigned correct priority	349 (55%)	309 (48%)
Calls assigned wrong priority	285 (45%)	335 (52%)

*p* = 0.012

**Table 2:** Sensitivity, specificity, PPV and NPV^a^ stratified by priority level using Medical Index and RETTS-A (*n* = 1278)

Priority level	Sensitivity	Specificity	PPV	NPV
Medical Index (*n* = 634)				
1	82.6% (76.6–87.3)	59.0% (54.3–63.5)	47.2% (76.6–87.3)	52.8% (47.5–58.0)
2	49.7% (42.2–52.5)	38.7% (34.3–43.4)	66.1%(60.3–71.4)	33.9% (28.6–39.7)
3	14.1% (8.0–24.0)	98.9% (97.7–99.5)	62.5% (38.6–81.5)	37.5% (18.5–61.4)
RETTS-A (*n* = 644)				
Red	54.0% (44.3–63.4)	83.6% (80.3–86.5)	37.8% (30.2–45.9)	62.2% (54.1–69.8)
Red+Orange^b^	78.9% (74.9–82.5)	48.0% (41.1–54.9)	77.4% (73.3–81.0)	22.6% (19.0–26.7)
Orange	53.2% (47.9–53.9)	57.0% (51.4–62.5)	59.0% (53.4–64.3)	41.0% (35.7–46.6)
Yellow	45.5% (37.6–53.6)	82.6% (79.0–85.6)	48.4% (40.6–56.2)	56.9% (48.9–64.5)
Green	9.4% (4.1–2.3)	95.1% (93.0–96.6)	13.9% (6.1–28.7)	86.1% (71.3–93.9)

95% confidence intervals in ()

^a^Sensitivity was calculated as true positives/(true positives + false negatives); Specificity as true negatives/(false positives + true negatives); Positive predictive value (PPV) as true positives/(true positives + false positives); Negative predictive value (NPV) as true negatives/(true negatives + false negatives)

^b^Alternative analysis where RETTS-A was converted from a four graded to a three graded priority protocol. The conversion was made by merging the two highest priority levels in to one single priority level (red + orange)

**Table 3:** Proportion of total over- and under triage^a^ using Medical Index and RETTS-A (*n* = 1278)

	Dispatch protocol	
	Medical Index (*n* = 634)	RETTS-A (*n* = 644)
Over triage	37.9% (34.2–41.7)	28.6% (25.2–32.2)
Under triage	6.3% (4.7–8.5)	23.4% (20.3–26.9)

95% confidence intervals in ()

^a^Over triage was defined as the assigned priority level being higher than the gold standard, under triage was defined as the assigned priority level being lower than the gold standard

In the alternative analysis where RETTS-A was converted to a three-graded priority protocol, the sensitivity for the highest priority level (red + orange) was 78.9% (Table 2) and total overall accuracy for priority level was 66% (*p* = 0.000) (Table 4).

**Table 4:** Alternative analysis^a^ of total accuracy in priority level using Medical Index and RETTS-A (*n* = 1278)

	Dispatch protocol	
	Medical Index (*n* = 634)	RETTS-A (*n* = 644)
Calls assigned correct priority	349 (55%)	423 (66%)
Calls assigned wrong priority	285 (45%)	221 (34%)

*p* = 0.000

^a^In the alternative analysis RETTS-A was converted from a four graded to a three graded priority protocol. The conversion was made by merging the two highest priority levels in to one single priority level (red + orange)

According to the gold standard of medical condition for each case, *n* = 492 (76.2%) were assessed correctly using the Medical Index and *n* = 457 (70.6%) using RETTS-A (*p* = 0.03). The median age of the participating telecommunicators was 42.5 (IQR: 16.25) and they had a median of seven years’ work experience (IQR: 6.0) at SOS Alarm (demographic data missing for 16 call takers). Eight of the 53 participating telecommunicators were RNs.

## Discussion

Our main results demonstrate that the Medical Index, the dispatch protocol currently in use, had a higher accuracy, measured as correctly assigned priority level, when compared with the newly developed RETTS-A. The sensitivity for the highest priority level was higher for the Medical Index as compared with RETTS-A. Despite the Medical Index being superior to RETTS-A, the overall accuracy of both protocols is to be considered as low. Comparisons are challenging, since RETTS-A has not previously been studied, and research on performance of accuracy in the Swedish Medical Index is limited [14, 15]. Even though differences exist in the dispatch process and pre-hospital setting [20], studies from Denmark and Norway are available for comparison, both using a CBD-protocol very similar to the Swedish Medical Index [5, 6, 21, 22]. In a small study from the sparsely populated County of Jämtland, Sweden, the sensitivity for dispatch priority level 1 and 2 was found to be to 94.5% [14]. This is higher than the corresponding sensitivity in the current study in both the Medical Index and RETTS-A. However, the authors merged priority level 1 and 2 into one single level in the Medical Index and used the three highest priority levels in the ambulance triage scale as the gold standard [14]. In our opinion, it may not be correct, from a patient safety perspective, to merge these levels, since the nature of the medical emergency and the timely need for medical assistance vary considerably between the merged levels. In another study of the Medical Index, a proportion of 27 and 53% of the priority level 1 and 2 calls was reported to be in accordance with the assessment by the ambulance at the scene (representing an overtriage of 76% and 18% respectively) [15]. Our results of high sensitivity for priority level 1 in the Medical Index are consistent with those reported from Denmark, where emergency medical calls with a high risk of hospital admission and death are assigned the highest priority level by the Danish Medical Index [5].

RETTS-A is based on the triage scale RETTS, but there is little scientific support for this system. To our knowledge, only two studies have been published [23, 24]. The authors suggested RETTS to be a triage scale with high correlation between assigned priority and the risk for mortality at the ED as well as following the hospital stay [23]. They also concluded that RETTS was a sensitive tool with which to detect patients with both high and low medical risk at the emergency department [24]. However, a systematic review evaluating the evidence for validity in triage scales stated that “the scientific evidence was found to be insufficient to assess the validity of RETTS” [25]. We believe our results with specified sensitivities for all priority levels in both protocols add an additional dimension to prior findings and contribute to a more nuanced picture.

The proportion of overtriage for both Medical Index and RETTS-A is substantially lower than that reported from Switzerland [7]. The difference may be explained by the different gold standards that were used. In our study, the expert consensus was based on the assessment of the information provided at the time of dispatching. A comparison with an assessment made later, i.e. in the ambulance would not be a true reflection of the assessment at the time of assessment by the telecommunicator in the EMCC. The Norwegian Medical Index has been found to have an association between priority level at dispatch and the need for pre-hospital medical care with a high sensitivity for low priority levels [21]. However, a high rate of overtriage occurred. The authors claim this validates the Norwegian Index as being good at “predicting those patients not in need of immediate medical treatment” [21]. This is in line with the current results of 6% under-triage and a specificity of 99% for the lowest priority level, indicating that it is a valid statement even for the Swedish version. The proportion of under-triage found in the previously mentioned Swiss study was similar to that of the Medical Index [7]. There is a lack of evidence on whether CBD affects clinical outcome [8], and there is no consensus on what levels of over- and undertriage are acceptable or desirable for dispatching. Guidelines for Trauma triage stipulate an acceptable range of overtriage of between 25 and 35% and undertriage of between 1 and 5%, depending on method and definition of gold standard [26]. This implies that an undertriage of 28%, as was seen for RETTS-A, should be viewed as a potential threat to patient safety.

The accuracy of assigned medical conditions was analysed and showed a higher accuracy for the Medical Index than RETTS-A. As described in the methods section, telecommunicators can assign two ESS’s to each call using RETTS-A, whilst in the Medical Index only one option is eligible. Despite this statistical advantage, the accuracy was lower in RETTS-A as compared to the Medical Index. RETTS-A is based on a triage scale that is developed for personnel with health care education, and uses medical terminology. The telecommunicators were supported with a list of clinical expressions used in the ESS-flowcharts with the aim to minimize the effect of the unfamiliarity with RETTS A and hence reduce the difference between the two tools. Since only eight of the 53 participating telecommunicators were RN’s, a lack of language comprehension might partly explain the inferiority in RETTS-A. However, a minority of telecommunicators at SOS Alarm are RN’s, so the participating cohort are representative of today’s situation. To our knowledge, no previous data on total accuracy for medical condition in the Swedish Medical Index is available for comparison [14, 15, 27]. Although correct priority level is the overriding factor for a dispatch protocol, a correct assessment of the medical condition is valuable, since the ambulance crew prepare themselves, both mentally and practically, based on the information in the dispatching [28]. Additionally, physician-staffed EMS-teams are commonly used in different EMS-systems to be dispatched for a subset of calls of medical-, surgical- and trauma-nature [29], and there is a trend that the response team may have unique equipment based on the medical condition, e.g. CT for suspected stroke [30]. Moreover, telecommunicators give pre-arrival instructions to callers based on medical condition until an ambulance arrives [31, 32]. Taken together, dispatch protocol should also perform with high accuracy for an assigned medical condition in order to direct the correct resource to the correct patient so as to further improve patient outcome.

### Limitations and future research

Dispatch research faces a number of challenges, mainly based on the lack of consensus on a set of criteria for evaluations and validation of dispatch [1, 11]. Comparative analysis of different dispatch protocols remains challenging, since both the type of protocol as well as the gold standard varies in the literature [33]. In the present study, we chose an expert consensus that was reached based on the written manuscripts as the gold standard. Depending on what questions the telecommunicator asked, different information in the manuscripts might have been provided by the standard patient, potentially creating a discrepancy in the information upon which the assessment was made by the call taker and the expert group. On the other hand, this reflects the real life of emergency medical dispatching, and these conditions were the same in both study groups and should, therefore, not have affected the comparison between the two dispatch protocols. The difference in accuracy and rates of over- and undertriage of the two protocols reported here could in part be explained by the fact that all participating telecommunicators had previous work experience in the Medical Index, whilst none of them were familiar with RETTS-A prior to the study. Another limitation is that we cannot present detailed data on each telecommunicator’s degree of adherence to the individual protocol.

In addition, the fact that the Medical Index is a three-graded priority protocol is a statistical advantage as compared with the four-graded protocol RETTS-A. However, the alternative analysis conversely demonstrated a higher overall accuracy for priority level in RETTS-A, and a less pronounced difference with respect to sensitivity for the highest priority level. On another hand, when merging the two highest priority levels into one, it creates an advantage for RETTS-A since any of these two levels is considered as a correct assessment when compared to the gold standard in the alternative analysis. Another factor potentially affecting the accuracy in dispatch research is the proportions of included calls as defined to each priority level by the reference, so that a higher accuracy of the protocol studied will be achieved if many obvious priority 1 calls are included. The current study included 26 scenarios, representing the six most common complaints to EMCCs. We deliberately excluded scenarios being too evidently of a priority 1 nature. The distribution of calls at each priority level is somewhat similar to that reported in epidemiology studies and dispatch research [5, 7, 15, 21, 34]. This suggests that our material is of clinical relevance, contributing to its generalizability.

Concerns have been raised that performance on written scenarios does not accurately mirror performance in practice [35]. Study designs based on fictitious cases rather than patients in a real-life setting have been stated to be suboptimal [25]. To further evaluate and improve the deliverance of health care in emergency medicine, investigation of real triage situations rather than scenarios is needed. Standard patients are commonly used in studies on medical communication and in certification/licensing [19], and have been reported to perform patient presentation with moderate to high levels of accuracy [36, 37] with the highest accuracy achieved for the history and management cases [36, 38, 39]. Their performance over time has been shown to be generally consistent without any evidence of the effect on warm-up or fatigue [39, 40]. The results suggest that standard patients can mimic a real-life emergency medical call, which overcomes the draw backs of written scenarios that do not allow for the interactions that can be gained in the interview situation. Hence, the current study also presents an innovative way of evaluating accuracy in emergency medical dispatching.

## Conclusion

In this simulation study we demonstrate that Medical Index had a higher accuracy for priority level and less undertriage than the new prototype for dispatch protocol, RETTS-A. Despite Medical Index being the superior tool, the overall accuracy of both protocols is to be considered as low. Overtriage challenges resource utilization while undertriage threatens patient safety. The results suggest that in order to improve patient safety both protocols need revisions in order to guarantee safe emergency medical dispatching.

## Abbreviations

CBD: Criteria-based dispatch protocol
CI: Confidence intervals
EMCC: Emergency Medical Communication Centre
EMS: Emergency medical services
ESS: Emergency signs and symptoms
IQR: Inter quartile range
NPV: Negative predictive value
PPV: Positive predictive value
RETTS: Rapid Emergency Triage and Treatment System
RN: Registered nurse
